# The HIV-1 Subtype C Epidemic in South America Is Linked to the United Kingdom

**DOI:** 10.1371/journal.pone.0009311

**Published:** 2010-02-19

**Authors:** Tulio de Oliveira, Deenan Pillay, Robert J. Gifford, for the UK Collaborative Group on HIV Drug Resistance

**Affiliations:** 1 Africa Centre for Health and Population Studies, Nelson R. Mandela School of Medicine, University of KwaZulu-Natal, Durban, South Africa; 2 Department of Infection, University College London, London, United Kingdom; 3 Centres for Infection, Health Protection Agency, Colindale, United Kingdom; 4 Zoology Department, University of Oxford, Oxford, United Kingdom; University of California San Francisco, United States of America

## Abstract

**Background:**

The global spread of HIV-1 has been accompanied by the emergence of genetically distinct viral strains. Over the past two decades subtype C viruses, which predominate in Southern and Eastern Africa, have spread rapidly throughout parts of South America. Phylogenetic studies indicate that subtype C viruses were introduced to South America through a single founder event that occurred in Southern Brazil. However, the external route via which subtype C viruses spread to the South American continent has remained unclear.

**Methodology/Principal Findings:**

We used automated genotyping to screen 8,309 HIV-1 subtype C *pol* gene sequences sampled within the UK for isolates genetically linked to the subtype C epidemic in South America. Maximum likelihood and Bayesian approaches were used to explore the phylogenetic relationships between 54 sequences identified in this screen, and a set of globally sampled subtype C reference sequences. Phylogenetic trees disclosed a robustly supported relationship between sequences from Brazil, the UK and East Africa. A monophyletic cluster comprised exclusively of sequences from the UK and Brazil was identified and dated to approximately the early 1980s using a Bayesian coalescent-based method. A sub-cluster of 27 sequences isolated from homosexual men of UK origin was also identified and dated to the early 1990s.

**Conclusions:**

Phylogenetic, demographic and temporal data support the conclusion that the UK was a crucial staging post in the spread of subtype C from East Africa to South America. This unexpected finding demonstrates the role of diffuse international networks in the global spread of HIV-1 infection, and the utility of globally sampled viral sequence data in revealing these networks. Additionally, we show that subtype C viruses are spreading within the UK amongst men who have sex with men.

## Introduction

Founder effects, genetic drift and recombination associated with the global spread of HIV-1 infection have given rise to genetically distinct viral strains referred to as ‘subtypes’ and ‘circulating recombinant forms’ [1]. HIV-1 genetic diversity may impact on disease progression and response to antiretroviral therapy, and has implications for vaccine development [2]. It is therefore important to monitor changes in the genetic and geographic complexity of the HIV-1 epidemic, and to identify the processes that drive these changes.

Of the various HIV-1 strains that have been described, the most prevalent worldwide is subtype C [3]. First described in East and Southern Africa [4], infections with viruses belonging to (or partially derived from) subtype C are now prevalent in regions throughout the world, including India, China, and South America [3], [5], [6]. In many of the regions where it has been introduced, subtype C has overtaken other HIV-1 strains introduced at earlier times [6]–[9]. Notably, studies suggest that subtype C may acquire multi-drug resistance more rapidly than other HIV-1 subtypes [10], [11].

The rapid spread of subtype C in regions of South America - including Brazil, Argentina and Uruguay - has drawn particular attention [12]–[16]. Recent studies indicate that the South American subtype C epidemic likely derives from a single founder virus that entered the continent via Southern Brazil, and was derived from viral strains prevalent in East Africa [12], [13]. However, the external route via which this virus spread from East Africa to South America has remained mysterious.

In the United Kingdom (UK), the prevalence of subtype C has increased steadily since the early 1990s, and it now ranks as the second most prevalent HIV-1 subtype after subtype B [17]. The overwhelming majority of subtype C infections in the UK occur in individuals whose reported exposure risk is heterosexual contact, and who were likely infected in Southern or Eastern Africa [18]. However, in a previous analyses of HIV-1 genetic diversity [19], we observed that some subtype C isolates sampled within the UK exhibit high levels of genetic similarity to isolates obtained in South America. To explore this finding in greater detail, we screened 8,309 subtype C sequences sampled within the UK to identify isolates genetically linked to the South American epidemic. We then examined the genetic relationships of these isolates to subtype C isolates sampled worldwide.

## Methods

### Study Group and Reference Sequences

8,309 subtype C *pol* gene sequences sampled within the UK were obtained from the UK HIV Drug Resistance Database (www.hivrdb.org.uk). These sequences were generated by population sequencing from plasma samples collected between 1996 and 2008, and were anonymously linked to data (obtained under voluntary agreement of patients) describing the ethnicity, nationality (country of birth) and exposure risk group of infected individuals. Sequences were at least 1000 nucleotides in length, spanning the genomic region between 2,253 and 3,251 nucleotides (HXB2 coordinates). Sequences are available on request from the UK HIV Drug Resistance Database.

A globally sampled reference sequence set comprising 1,289 previously published subtype C *pol* gene sequences annotated by country of sampling was obtained from the Los Alamos HIV Sequence Database (www.hiv.lanl.gov). The reference set included sequences from Argentina (n = 8), Burundi (n = 92), Brazil (n = 122), Botswana (n = 144), Ethiopia (n = 101), India (n = 74), Kenya (n = 3), Tanzania (n = 65), Uganda (n = 11), South Africa (n = 667) and the UK (n = 2). The IDs of reference sequences used in this study are provided as supplementary information (File S1).

### Sequence Analysis

Sequences were classified into phylogenetic groups (i.e. subtypes, circulating recombinant forms and within-subtype lineages) using the REGA HIV-1 subtyping tool (version 2.0, available at: www.bioafrica.net) [20]–[22]. Sequence alignments were created using MUSCLE [23] and manually edited. Maximum likelihood phylogenies were constructed using PhyML [24] and parameters estimated from the dataset (nucleotide substitution model = HKY85, transition/transversion ratio = 4.0, gamma shape parameter = 0.780). Bayesian phylogenetic analysis was performed using MrBayes v3.1.2 [25]. Bayesian phylogenies were inferred using the GTR+I+Γ nucleotide substitution selected using Modeltest [26]. For each dataset, two runs (one cold and one tree heated, temp¼0.20) of four chains each were run for 10^7^ generations, with trees sampled every 1000^th^ generation. The burn-in of 10% was excluded from the analysis. Convergence of parameters was assessed by calculating the effective sample size (ESS) using TRACER v1.4 [27], excluding an initial 10% for each run. All parameters estimates for each run showed ESS values more than 300. Shared drug resistance mutations were identified using the calibrated population resistance (CPR) tool [28].

### Estimation of Evolutionary Rates and Dates

All sequences used for estimation of dates were examined for evidence of inter- and intra-subtype recombination. Sequences that were not classified as pure (non-recombinant) subtype C by REGA (inter-subtype recombination) [21], [22] and SCUEL (intra-subtype recombination) [29] were excluded. Estimates of the evolutionary rate and the date of the most recent common ancestor (MRCA) were performed using a Bayesian Markov chain Monte Carlo (MCMC) approach as implemented in BEAST v1.7. Analyses were performed with a Bayesian Skyline coalescent tree prior, under the GTR + I + Γ model of nucleotide substitution, and using both a strict and a relaxed molecular clock (uncorrelated Lognormal model). Two separate MCMC chains were run for 10^8^ generations for each dataset, sampled every 10,000^th^ generation. BEAST output was analyzed using TRACER v1.4 [27], with uncertainty in parameter estimates reflected in the 95% highest probability density (HPD) values after excluding a burn-in of 10%.

## Results

An automated genotyping procedure [21], [22] was used to screen 8,309 subtype C *pol* gene sequences from the UK for isolates genetically linked to the subtype C epidemic in South America (data not shown). Screening identified a minority of sequences (n = 54, <1%) potentially linked to the South American subtype C epidemic. These 54 sequences were aligned with a set of 1,289 globally sampled reference sequences, plus 84 additional sequences from the UK. A maximum likelihood (ML) phylogeny constructed using this alignment confirmed the relationship between the 54 UK sequences and South American isolates, disclosing a well-supported monophyletic group comprised exclusively of subtype C *pol* genes sampled in East Africa, Brazil and the UK (Figure S1, Figure S2). Notably, sequences from the UK were intermingled with sequences from Brazil within this clade. Previously, no isolate obtained outside South America has been reported to group within the monophyletic lineage defined by South American subtype C isolates.

To explore these relationships in greater depth, we conducted a detailed Bayesian Markov chain Monte Carlo (MCMC) phylogenetic analysis using all 54 UK sequences identified by screening, and a representative set of 207 reference sequences from Africa (n = 110), Asia (n = 10) and Brazil (n = 87). The Bayesian skyline and relaxed clock models were selected over demographic (constant and exponential growth) and strict molecular clock models using Bayes factor analysis [30] (support >20 at log10 Bayes factor scale). The mean mutation rate for the Bayesian skyline relaxed clock model was estimated as 1.81×10^−3^ nucleotide substitutions per site per year (HPDs 1.27 to 2.37×10^−3^), within the range of rates obtained for approximately equivalent genomic regions in previous studies (Table S1). All Bayesian MCMC independent runs converged to almost identical values for all parameters, and the ESS values for estimates were more than 300.

**Figure 1:**
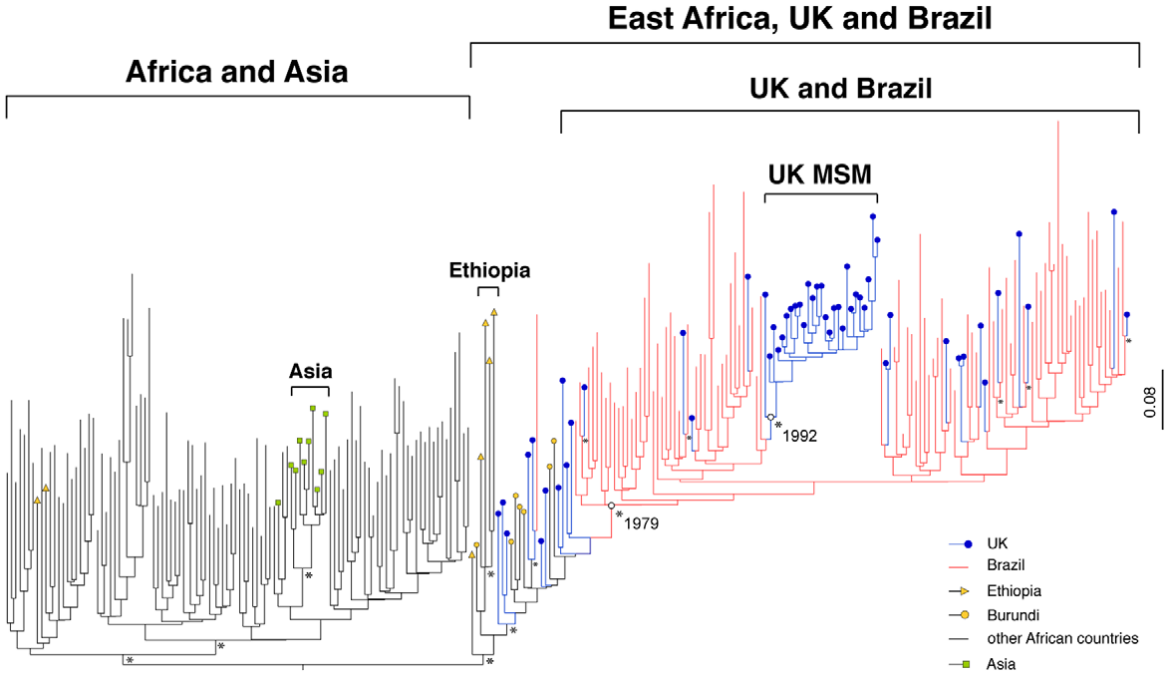
Bayesian tree of HIV-1 subtype C *pol* sequences. Evolutionary relationships between 54 United Kingdom, 87 Brazilian and 120 non-Brazilian subtype C *pol* sequences, estimated using Bayesian phylogenetic analysis, are shown. Colors on terminal branches and terminal nodes indicate the geographic location of sampling, as shown in the key (bottom right). Asterisks indicate nodes with posterior probability values of 0.95 or higher. Brackets indicate clades comprised of sequences sampled from a specific geographic region, and with posterior probability values above 0.95. The mean dates of the most recent common ancestors to the ‘UK and Brazil’ and ‘UK-MSM’ clades are indicated alongside the corresponding internal nodes (white circles). Note that the majority (n = 8,266, >99%) of the UK subtype C sequences examined in this report grouped within the ‘Africa and Asia’ clade in preliminary analysis and are not shown here.

As shown in Figure 1, Bayesian trees supported the grouping of East African, UK and Brazilian isolates into a single monophyletic cluster. Within this cluster, East African isolates (from Ethiopia and Burundi) grouped basally, whereas Brazilian and UK isolates occupied more derived positions. A total of 43 sequences from the UK grouped together with 86 Brazilian sequences to form a single monophyletic clade, while a smaller number of UK sequences (n = 11) grouped basally with respect to this ‘UK-Brazil’ clade (Figure 1). Amongst the eleven UK sequences positioned basal to the UK-Brazil clade, those positioned nearer the tree root were obtained from East African immigrants, whereas those occupying more derived positions were obtained from individuals born outside Africa.

Within the UK-Brazil clade, sequences from both geographic regions were generally intermixed. However, 27 UK sequences formed a single well-supported group displaying short branch lengths suggestive of a local transmission chain [31]. Exposure risk and ethnicity were reported for approximately half (n = 15) of these infections; revealing that all were obtained from Caucasians, the majority of whom (87%, n = 13) were men who have sex with men (MSM). Country of birth was reported for eleven individuals in this ‘UK-MSM’ cluster, revealing that seven (64%) were born in the UK. Overall these data stand in contrast to the majority of subtype C sequences from the UK for which similar data were available (n = 2332), most of which were obtained from individuals who were African by ethnicity and country of birth (73%), and/or reported their exposure risk as heterosexual sex (84%).

The grouping of subtype C sequences into ‘East Africa-UK-Brazil’, ‘UK-Brazil’, and ‘UK-MSM’ clusters was supported by high posterior probabilities (>0.95) in each case (Figure 1), and was robust to the exclusion of positions in the reverse transcriptase gene at which shared drug resistance mutations were present (positions 85, 103, 184, 190, 215).

We used a Bayesian MCMC approach to estimate the dates of most recent common ancestors (MRCA) for the UK-Brazil, and UK MSM clades. For the UK-Brazil clade the estimated time of the MRCA was 1980 (1972 to 1987) under a “relaxed molecular clock” model and 1977 (1969 to 1985) under a “strict clock” model. These estimates approximately correspond to those previously calculated for the MRCA of the South American epidemic [12]. For the UK MSM clade we estimated an origin in 1992 (1988–1997) using a relaxed clock model, and 1990 (1985 to 1994) using a strict clock model. Alternative demographic models gave comparable results (data not shown).

## Discussion

The data presented here provide clear evidence of an epidemiological link between subtype C epidemics in East Africa, South America, and the UK. While previous studies of the subtype C epidemic in South America have indicated a link to East Africa [12], the nature of this link has remained mysterious, particularly since social, cultural, and economic relationships between the two regions are limited. The UK fits well as the missing piece in this puzzle. Firstly, it is home to large Brazilian and East African immigrant populations (i.e. >100,000 individuals), both of which are concentrated in London [32], [33]. Furthermore, these populations are skewed toward adults between the ages of 20 and 30, a demographic likely to be involved in high-risk behaviors associated with transmission of HIV-1 [34]. It should also be noted that both Brazil and East Africa are reported destinations for UK ‘sex tourism’ (defined as travel specifically for the purpose of engaging in sexual activity) [35]. All of these factors can be considered likely to have played a role in the emergence of linked subtype C epidemics in the three distinct geographic regions.

The African origin of HIV-1 is now well established [36]. Assuming that the reference sequences used in this analysis provide an adequate representation of global subtype C diversity, our data support a scenario under which East African subtype C strains were introduced first to the UK, and subsequently to Brazil. Although alternative scenarios cannot be conclusively ruled out by our analysis, transfer from East Africa to Brazil via the UK is consistent with cultural and demographic data (see above) as well with the phylogenetic and temporal structuring of infections (Figure 1). The intermingling of sequences from the UK and Brazil in phylogenies also raises the possibility that multiple transfers of subtype C viruses between the UK and Brazil (in both directions) may have occurred in recent times.

The identification of a well-supported and relatively large cluster of subtype C infections in Caucasian males, the majority of whom were born in the UK and reported their exposure risk as sex between men, suggests that subtype C is spreading amongst MSM within the UK. This observation reinforces previous reports that the HIV-1 epidemic in UK MSM, which is historically associated with subtype B viruses, is diversifying [19]. The estimated time of the MRCA for the cluster of subtype C infections identified in UK MSM (Figure 1) approximately corresponds to that estimated for a cluster of subtype A infections identified previously in the same exposure risk population (1989–1994) [19]. Together, these data indicate that diverse non-subtype B strains have been circulating amongst MSM in the UK for nearly two decades, sufficient time for infections to have spread within this population and, potentially, to epidemiologically linked populations throughout Europe [37]. The increasing diversity of the HIV-1 epidemic amongst UK MSM may have implications for future diagnosis, treatment and prevention in this exposure risk group [2].

The data presented here reveal a novel perspective on the origin and evolutionary history of the subtype C epidemic in South America, and emphasize the role of diffuse international networks in the global dissemination of HIV-1. In addition, this analysis further demonstrates the utility of globally sampled viral sequence data in unraveling the complex routes by which sexually transmitted infections spread across international borders.

## Supporting Information

Table S1 (0.06 MB DOC)

Figure S1 Maximum likelihood phylogenetic tree based on 999 nucleotides of pol gene sequence (nucleotides 2,253–3,251 (HXB2 coordinates)) from 1,427 HIV-1 subtype C isolates. Sequences were isolated in the following countries; Argentina (n = 8); Burundi (n = 92); Brazil (n = 122); Botswana (n = 144); Ethiopia (n = 101); India (n = 74); Kenya (n = 3); Tanzania (n = 65); Uganda (n = 11); South Africa (n = 667); UK (unpublished sequences n = 138) (total n = 140). Monophyletic clusters are marked as follows; grey (African cluster); yellow (Indian cluster); brown (Ethiopia/Burundi cluster); green (Burundi/UK cluster); blue (UK/Brazilian cluster). Tip branches representing UK sequences are colored white. Marked clusters showed aLRT support >0.7.
(0.12 MB PDF)

Figure S2 Detailed maximum likelihood phylogenetic tree showing tip and node aLRT values, and constructed using 999 nucleotides of pol gene sequence (nucleotides 2,253–3,251 (HXB2 coordinates)) from 1,427 HIV-1 subtype C isolates. Sequences were isolated in the following countries; Argentina (n = 8); Burundi (n = 92); Brazil (n = 122); Botswana (n = 144); Ethiopia (n = 101); India (n = 74); Kenya (n = 3); Tanzania (n = 65); Uganda (n = 11); South Africa (n = 667); UK (unpublished sequences n = 138) (total n = 140). Monophyletic clusters are marked as follows; grey (African cluster); yellow (Indian cluster); brown (Ethiopia/Burundi cluster); green (Burundi/UK cluster); blue (UK/Brazilian cluster). Tip branches representing UK sequences are colored white. Marked clusters showed aLRT support >0.7.
(4.88 MB PDF)

File S1 (0.09 MB DOC)
