# Supplementary material for: The HIV-1 Subtype C Epidemic in South America Is Linked to the United Kingdom
Source: PLoS One. 2010 Feb 19;5(2):e9311. doi: 10.1371/journal.pone.0009311 (PMC2824804; doi:10.1371/journal.pone.0009311)
Supplement: Table S1 — (0.06 MB DOC) [file pone.0009311.s001.doc]

**Table S1.** Comparison of evolutionary rates estimated for the HIV-1 *pol* gene

| **Manuscript** | **Mean Rate [lower ad upper highest posterior density intervals]** | **Subtype** |
| --- | --- | --- |
| Salemi *et al*. PLoS One 2008 [1] | 1.4 x 10^-3^ (strict clock) [0.28 - 2.6] | Subtype A |
| Hue *et al*. J Virology 2009 [2] | 2.6 x 10^-3^ (strict clock) [1.9 – 3.3] | Subtype B |
| Hue *et al*. PNAS 2005 [3] | 2.55 x 10^-3^ (strict clock) [1.7 - 3.5] | Subtype B |
| Dalai *et el.* AIDS 2009 [4] | 2.16 x 10^-3^ (strict clock) [1.8 - 2.5]  2.19 x 10^-3^ (relax clock) [1.7 - 2.5] | Subtype C |
| Bello *et al.* AIDS 2008 [5] | 1.5 x 10^-3^ (strict clock) [1.0 - 2.0]  1.6 x 10^-3^ (relax clock) [1.0 – 2.0] | Subtype C |
| Tee *et al.* J. Virology 2008 [6] | 1.8 x 10^-3^ (strict clock) [1.4 – 2.3] | Subtype C |
| De Oliveira *et al*, (this manuscript) | 1.64 x 10^-3^ (strict clock) [1.18 - 2.09]  1.81 x 10^-3^ (relax clock) [1.27 - 2.37] | Subtype C |

**Footnote:** All rates were estimated for the region between 2,253 and 3,251 nucleotides (HXB2 coordinates), and extending no more than 500 nucleotides beyond this range in either direction.

**References**

1. Salemi M, de Oliveira T, Ciccozzi M, Rezza G, Goodenow MM (2008) High-resolution molecular epidemiology and evolutionary history of HIV-1 subtypes in Albania. PLoS One 3: e1390.

2. Hue S, Gifford RJ, Dunn D, Fernhill E, Pillay D (2009) Demonstration of sustained drug-resistant human immunodeficiency virus type 1 lineages circulating among treatment-naive individuals. J Virol 83: 2645-2654.

3. Hue S, Pillay D, Clewley JP, Pybus OG (2005) Genetic analysis reveals the complex structure of HIV-1 transmission within defined risk groups. Proc Natl Acad Sci U S A 102: 4425-4429.

4. Dalai SC, de Oliveira T, Harkins GW, Kassaye SG, Lint J, et al. (2009) Evolution and molecular epidemiology of subtype C HIV-1 in Zimbabwe. AIDS 23: 2523-2532.

5. Bello G, Passaes CP, Guimaraes ML, Lorete RS, Matos Almeida SE, et al. (2008) Origin and evolutionary history of HIV-1 subtype C in Brazil. AIDS 22: 1993-2000.

6. Tee KK, Pybus OG, Li XJ, Han X, Shang H, et al. (2008) Temporal and spatial dynamics of human immunodeficiency virus type 1 circulating recombinant forms 08_BC and 07_BC in Asia. J Virol 82: 9206-9215.
