# Supplementary figures and images for: The HIV-1 Subtype C Epidemic in South America Is Linked to the United Kingdom
Source: PLoS One. 2010 Feb 19;5(2):e9311. doi: 10.1371/journal.pone.0009311 (PMC2824804; doi:10.1371/journal.pone.0009311)

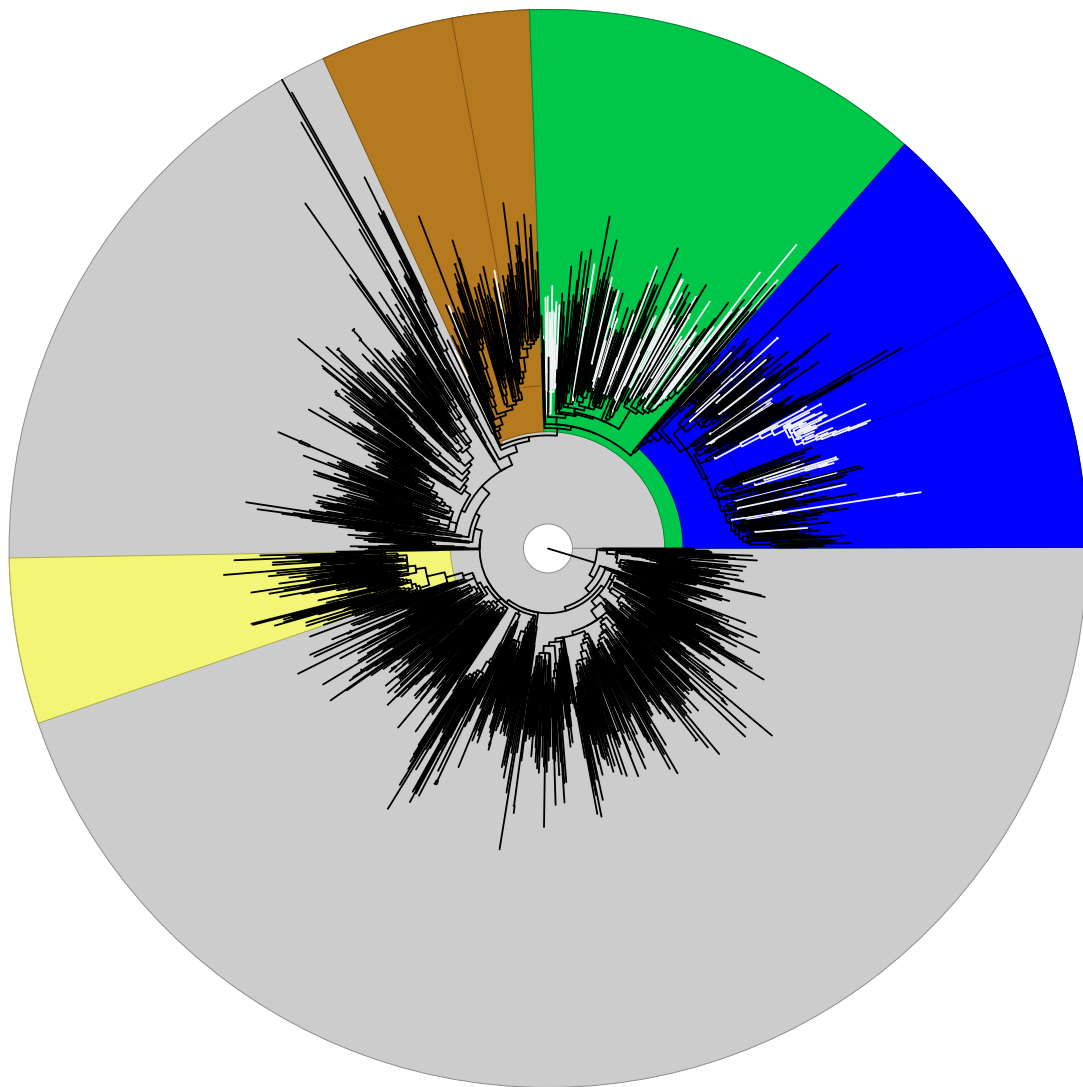

0.05

Supplement: Figure S1 — Maximum likelihood phylogenetic tree based on 999 nucleotides of pol gene sequence (nucleotides 2,253–3,251 (HXB2 coordinates)) from 1,427 HIV-1 subtype C isolates. Sequences were isolated in the following countries; Argentina (n = 8); Burundi (n = 92); Brazil (n = 122); Botswana (n = 144); Ethiopia (n = 101); India (n = 74); Kenya (n = 3); Tanzania (n = 65); Uganda (n = 11); South Africa (n = 667); UK (unpublished sequences n = 138) (total n = 140). Monophyletic clusters are marked as follows; grey (African cluster); yellow (Indian cluster); brown (Ethiopia/Burundi cluster); green (Burundi/UK cluster); blue (UK/Brazilian cluster). Tip branches representing UK sequences are colored white. Marked clusters showed aLRT support >0.7. (0.12 MB PDF) [file pone.0009311.s002.pdf]

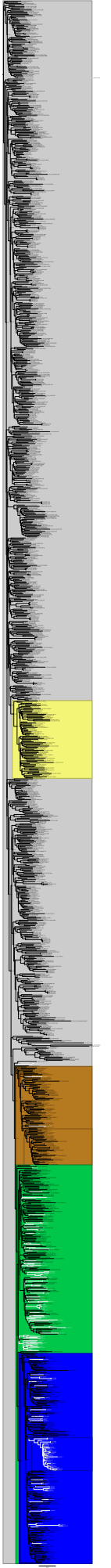

Supplement: Figure S2 — Detailed maximum likelihood phylogenetic tree showing tip and node aLRT values, and constructed using 999 nucleotides of pol gene sequence (nucleotides 2,253–3,251 (HXB2 coordinates)) from 1,427 HIV-1 subtype C isolates. Sequences were isolated in the following countries; Argentina (n = 8); Burundi (n = 92); Brazil (n = 122); Botswana (n = 144); Ethiopia (n = 101); India (n = 74); Kenya (n = 3); Tanzania (n = 65); Uganda (n = 11); South Africa (n = 667); UK (unpublished sequences n = 138) (total n = 140). Monophyletic clusters are marked as follows; grey (African cluster); yellow (Indian cluster); brown (Ethiopia/Burundi cluster); green (Burundi/UK cluster); blue (UK/Brazilian cluster). Tip branches representing UK sequences are colored white. Marked clusters showed aLRT support >0.7. (4.88 MB PDF) [file pone.0009311.s003.pdf]
