# Supplementary material for: The HIV-1 Subtype C Epidemic in South America Is Linked to the United Kingdom
Source: PLoS One. 2010 Feb 19;5(2):e9311. doi: 10.1371/journal.pone.0009311 (PMC2824804; doi:10.1371/journal.pone.0009311)
Supplement: File S1 — (0.09 MB DOC) [file pone.0009311.s004.doc]

**File S1: Sequence alignment details.** Maximum likelihood phylogenies shown in supplementary figures 1 and 2 were constructed using an alignment comprised of 1,427 sequences, of which 1,289 were obtained from the Los Alamos HIV Sequence Database ([www.hiv.lanl.gov](http://www.hiv.lanl.gov/)), and 138 from the UK HIV Drug Resistance Database ([www.hivrdb.org.uk](http://www.hivrdb.org.uk/)). Sequence IDs used in the alignment are listed below. Los Alamos sequence IDs follow the standard format of “subtype.country.year_of_isolation.isolate_name” (example: C.AR.2001.96105: subtype=C, country=AR (Argentina), isolation year, 96105 – isolate name). Alignments are available on request from the authors.

**Los Alamos HIV database sequence IDs**

C.AR.2001.96105 ; C.AR.2001.ARG4005 ; C.AR.2001.ARG4006 ; C.AR.2002.ARG4009 ; C.AR.2003.F007 ; C.AR.2004.F098 ; C.AR.2005.F349 ; C.AR.2005.F379 ; C.BI.2002.02BUB1027 ; C.BI.2002.02BUB1040 ; C.BI.2002.02BUB1048 ; C.BI.2002.02BUB1051 ; C.BI.2002.02BUB1054 ; C.BI.2002.02BUB1075 ; C.BI.2002.02BUB1078 ; C.BI.2002.02BUB1085 ; C.BI.2002.02BUB1099 ; C.BI.2002.02BUB1100 ; C.BI.2002.02BUB1104 ; C.BI.2002.02BUSF130 ; C.BI.2002.02BUSF213 ; C.BI.2002.02BUSF302 ; C.BI.2002.02BUSF426 ; C.BI.2002.02BUSF428 ; C.BI.2002.02BUSF431 ; C.BI.2002.02BUSF621 ; C.BI.2002.02BUU0118 ; C.BI.2002.02BUU0126 ; C.BI.2002.02BUU0127 ; C.BI.2002.02BUU0139 ; C.BI.2002.02BUU0211 ; C.BI.2002.02BUU0221 ; C.BI.2002.02BUU0304 ; C.BI.2002.02BUU0333 ; C.BI.2002.02BUU0427 ; C.BI.2002.02BUU0434 ; C.BI.2002.02BUU0435 ; C.BI.2002.02BUU0505 ; C.BI.2002.02BUU0636 ; C.BI.2002.02BUU0702 ; C.BI.2002.02BUU0705 ; C.BI.2002.02BUU0710 ; C.BI.2002.02BUU0727 ; C.BI.2002.02BUU0735 ; C.BI.2002.02BUU0804 ; C.BI.2002.02BUU0808 ; C.BI.2002.02BUU0812 ; C.BI.2002.02BUU0813 ; C.BI.2002.02BUU0820 ; C.BI.2002.02BUU0824 ; C.BI.2002.02BUU0826 ; C.BI.2002.02BUU0832 ; C.BI.2002.02BUU0932 ; C.BI.2002.02BUU1005 ; C.BI.2002.02BUU1029 ; C.BI.2002.02BUU1101 ; C.BI.2002.02BUU1133 ; C.BI.2002.02BUU1206 ; C.BI.2002.02BUU1305 ; C.BI.2002.02BUU1327 ; C.BI.2002.02BUU1333 ; C.BI.2002.02BUU1506 ; C.BI.2002.02BUU1507 ; C.BI.2002.02BUU1516 ; C.BI.2002.02BUU1605 ; C.BI.2002.02BUU1620 ; C.BI.2002.02BUU1622 ; C.BI.2002.02BUU1625 ; C.BI.2002.02BUU1703 ; C.BI.2002.02BUU1732 ; C.BI.2002.02BUU1734 ; C.BI.2002.02BUU1811 ; C.BI.2002.02BUU1817 ; C.BI.2002.02BUU1818 ; C.BI.2002.02BUU1825 ; C.BI.2002.02BUU1912 ; C.BI.2002.02BUU1930 ; C.BI.2002.02BUU2013 ; C.BI.2002.02BUU2025 ; C.BI.2002.02BUU2118 ; C.BI.2002.02BUU2132 ; C.BI.2002.02BUU2201 ; C.BI.2002.02BUU2202 ; C.BI.2002.02BUU2212 ; C.BI.2002.02BUU2231 ; C.BI.2002.02BUU2233 ; C.BI.2002.02BUU2234 ; C.BI.2002.02BUU2335 ; C.BI.2002.02BUU2417 ; C.BI.2002.02BUU2515 ; C.BI.2002.02BUU2517 ; C.BI.2002.02BUU2702 ; C.BI.2002.02BUU2809 ; C.BI.2002.02BUU2824 ; C.BI.2002.02BUU2905 ; C.BI.2002.02BUU2909 ; C.BI.2002.02BUU3124 ; C.BI.2002.02BUU3135 ; C.BI.2002.02BUU3210 ; C.BI.2002.02BUU3213 ; C.BR.00.RGS09 ; C.BR.00.RGS23 ; C.BR.00.RGS29 ; C.BR.00.RGS33 ; C.BR.01.RGS37 ; C.BR.01.RGS41 ; C.BR.01.RGS47 ; C.BR.01.RGS55 ; C.BR.01.RGS59 ; C.BR.01.RGS61 ; C.BR.01.RGS71 ; C.BR.02.HC29 ; C.BR.02.HC35 ; C.BR.02.HC37 ; C.BR.02.HF25 ; C.BR.02.HF45 ; C.BR.02.RGS83 ; C.BR.02.RGS89 ; C.BR.172 ; C.BR.1992.BR025d ; C.BR.1998.98BR004 ; C.BR.2001.BRC1526POL ; C.BR.2001.BRC1527POL ; C.BR.2001.BRC1550POL ; C.BR.2001.BRC1563POL ; C.BR.2002.038PRRT ; C.BR.2002.092PRRT ; C.BR.2002.B150 ; C.BR.2002.C059 ; C.BR.2002.C062 ; C.BR.2002.C073 ; C.BR.2002.C114 ; C.BR.2002.c135 ; C.BR.2002.C152 ; C.BR.2002.C160 ; C.BR.2002.PA211 ; C.BR.2002.PA244 ; C.BR.2002.PA254 ; C.BR.2002.PA255 ; C.BR.2002.PA257 ; C.BR.2002.PA261 ; C.BR.2002.PA269 ; C.BR.2002.PA341 ; C.BR.2004.04BR013 ; C.BR.2004.04BR021 ; C.BR.2004.04BR038 ; C.BR.2004.04BR073 ; C.BR.2004.74023 ; C.BR.2004.74039 ; C.BR.2004.74062 ; C.BR.2004.84004 ; C.BR.2004.84019 ; C.BR.2004.84021 ; C.BR.2004.84024 ; C.BR.2004.85012 ; C.BR.2004.85020 ; C.BR.2004.85034 ; C.BR.2004.86006 ; C.BR.2004.86009 ; C.BR.2004.86011 ; C.BR.2004.86012 ; C.BR.2004.86014 ; C.BR.2004.86043 ; C.BR.2004.86062 ; C.BR.2004.BR04PR63 ; C.BR.2004.BR04PR64 ; C.BR.2004.BR04SC80 ; C.BR.2005.BR05PR413 ; C.BR.2005.BR05PR428 ; C.BR.2005.BR05PR430 ; C.BR.2005.BR05PR448 ; C.BR.2005.BR05RS589 ; C.BR.2005.BR05RS778 ; C.BR.2005.BR05RS791 ; C.BR.2005.BR05SC300 ; C.BR.2005.BR05SC308 ; C.BR.2005.BR05SC316 ; C.BR.2005.BR05SC332 ; C.BR.2006.BR06PR38 ; C.BR.2006.BR06PR43 ; C.BR.2006.BR06PR48 ; C.BR.2006.BR06PR626 ; C.BR.2006.BR06PR631 ; C.BR.2006.BR06PR632 ; C.BR.2006.BR06PR635 ; C.BR.2006.BR06PR822 ; C.BR.2006.BR06PR826 ; C.BR.2006.BR06RS467 ; C.BR.2006.BR06RS484 ; C.BR.2006.BR06RS781 ; C.BR.2006.BR06RS802 ; C.BR.2006.BR06SC251 ; C.BR.2006.BR06SC253 ; C.BR.2006.BR06SC269 ; C.BR.2006.BR06SC277 ; C.BR.2006.BR06SC283 ; C.BR.2006.BR06SC284 ; C.BR.2006.BR06SC292 ; C.BR.2006.BR06SC293 ; C.BR.2006.BR06SC396 ; C.BR.2006.BR06SC399 ; C.BR.2006.BR06SC400 ; C.BR.2006.BRLUS2437 ; C.BR.98.RS010 ; C.BR.98.RS013 ; C.BR.98.RS022 ; C.BR.98.RS027 ; C.BR.98.RS046 ; C.BR.98.RS048 ; C.BR.98.RS049 ; C.BR.98.RS050 ; C.BR.98.RS059 ; C.BR.98.RS069 ; C.BR.98.RS075 ; C.BR.98.RS076 ; C.BR.98.RS089 ; C.BR.res037 ; C.BR.res079 ; C.BR.RES083 ; C.BR.RG033 ; C.BR.RS481 ; C.BR.tvgg41 ; C.BW.1996.96BW01B03 ; C.BW.1996.96BW0402 ; C.BW.1996.96BW0502 ; C.BW.1996.96BW06H51 ; C.BW.1996.96BW1104 ; C.BW.1996.96BW1210 ; C.BW.1996.96BW15B03 ; C.BW.1996.96BW15C05 ; C.BW.1996.96BW16B01 ; C.BW.1996.96BW17 ; C.BW.1996.96BWM032 ; C.BW.1996.96BWMO15 ; C.BW.1998.98BWMC122 ; C.BW.1998.98BWMC134 ; C.BW.1998.98BWMC14A3 ; C.BW.1998.98BWMO1410 ; C.BW.1998.98BWMO18D5 ; C.BW.1998.98BWMO36A5 ; C.BW.1998.98BWMO37D5 ; C.BW.1999.99BW393212 ; C.BW.1999.99BW46424 ; C.BW.1999.99BW4745 ; C.BW.1999.99BW47547 ; C.BW.1999.99BWMC168 ; C.BW.2000.00BW07621 ; C.BW.2000.00BW076820 ; C.BW.2000.00BW087421 ; C.BW.2000.00BW147127 ; C.BW.2000.00BW16162 ; C.BW.2000.00BW1686 ; C.BW.2000.00BW17593 ; C.BW.2000.00BW17732 ; C.BW.2000.00BW17835 ; C.BW.2000.00BW17956 ; C.BW.2000.00BW18113 ; C.BW.2000.00BW18595 ; C.BW.2000.00BW18802 ; C.BW.2000.00BW192113 ; C.BW.2000.00BW20361 ; C.BW.2000.00BW20636 ; C.BW.2000.00BW20872 ; C.BW.2000.00BW2127214 ; C.BW.2000.00BW22767 ; C.BW.2000.00BW38193 ; C.BW.2000.00BW38428 ; C.BW.2000.00BW38713 ; C.BW.2000.00BW38769 ; C.BW.2000.00BW38868 ; C.BW.2000.00BW38916 ; C.BW.2000.00BW39702 ; C.BW.2000.00BW50311 ; C.BW.2001.01BW0498S ; C.BW.2001.01BW0797N ; C.BW.2001.01BW0800N ; C.BW.2001.01BW0961S ; C.BW.2001.01BW0971S ; C.BW.2001.01BW0972S ; C.BW.2001.01BW1025N ; C.BW.2001.01BW1187S ; C.BW.2001.01BW1374S ; C.BW.2001.01BW1375S ; C.BW.2001.01BW1378S ; C.BW.2001.01BW1386S ; C.BW.2001.01BW1387S ; C.BW.2001.01BW1392S ; C.BW.2001.01BW1432N ; C.BW.2001.01BW1451N ; C.BW.2001.01BW1549S ; C.BW.2001.01BW1557S ; C.BW.2001.01BW1780S ; C.BW.2001.01BW1899S ; C.BW.2001.01BW1900S ; C.BW.2001.01BW1934S ; C.BW.2001.01BW2053S ; C.BW.2001.01BW2064S ; C.BW.2001.01BW2065S ; C.BW.2001.01BW2165S ; C.BW.2001.01BW2206N ; C.BW.2001.01BW2210N ; C.BW.2001.01BW2414S ; C.BW.2001.01BW2433N ; C.BW.2001.01BW2508S ; C.BW.2001.01BW2528S ; C.BW.2001.01BW2614N ; C.BW.2001.01BW2673S ; C.BW.2001.01BW2790S ; C.BW.2001.01BW2793S ; C.BW.2001.01BW2796S ; C.BW.2001.01BW2844N ; C.BW.2001.01BW3224N ; C.BW.2001.01BW3226N ; C.BW.2001.01BW3368N ; C.BW.2001.01BW3374N ; C.BW.2001.01BW3381N ; C.BW.2001.01BW3383N ; C.BW.2001.01BW3388N ; C.BW.2001.01BW3389N ; C.BW.2001.01BW3538S ; C.BW.2001.01BW3582N ; C.BW.2001.01BW3605N ; C.BW.2001.01BW3726N ; C.BW.2001.01BW3772S ; C.BW.2001.01BW3845N ; C.BW.2001.01BW3895N ; C.BW.2001.01BW3920N ; C.BW.2001.01BW3930N ; C.BW.2001.01BW3931N ; C.BW.2001.01BW4009S ; C.BW.2001.01BW4011S ; C.BW.2001.01BW4012S ; C.BW.2001.01BW4013S ; C.BW.2001.01BW4127N ; C.BW.2001.01BW4136N ; C.BW.2001.01BW4271N ; C.BW.2001.01BW4292N ; C.BW.2001.01BW4309N ; C.BW.2001.01BW4330N ; C.BW.2001.01BW4589N ; C.BW.2001.01BW4755N ; C.BW.2001.01BW4789N ; C.BW.2001.01BW4902N ; C.BW.B0008955GP00019 ; C.BW.B0008985AT07976 ; C.BW.B0010253GP00078 ; C.BW.B0010720AN88477 ; C.BW.B0011441GP00155 ; C.BW.B0011946GP00201 ; C.BW.B0011952GP00208 ; C.BW.B0012378BH72118 ; C.BW.B0012823BT20525 ; C.BW.B0014471GP00458 ; C.BW.B0014671GP00493 ; C.BW.B0014976GP00550 ; C.BW.B0015990AN20566 ; C.BW.B0016041BH55370 ; C.BW.B0016174BT19067 ; C.BW.B0016241AT17985 ; C.BW.B0016463GP03858 ; C.BW.B0017089BT25064 ; C.BW.B0018916BE30851 ; C.BW.B0018988BJ14401 ; C.BW.B0020517AN42459 ; C.BW.B0021311BH54154 ; C.BW.B0021466AR1455 ; C.ET.1986.ETH2220 ; C.ET.1988.AS88651GP ; C.ET.1988.DD88379GP ; C.ET.1988.DD88477GP ; C.ET.1988.DE88404GP ; C.ET.1988.GO88052GP ; C.ET.1996.AM96146GP ; C.ET.1996.AM96148GP ; C.ET.1996.JM96102GP ; C.ET.2000.s03928 ; C.ET.2002.02ET288 ; C.ET.2003.ETHG001 ; C.ET.2003.ETHG008 ; C.ET.2003.ETHG009 ; C.ET.2003.ETHG010 ; C.ET.2003.ETHG012 ; C.ET.2003.ETHG016 ; C.ET.2003.ETHG021 ; C.ET.2003.ETHG022 ; C.ET.2003.ETHG023 ; C.ET.2003.ETHG026 ; C.ET.2003.ETHG027 ; C.ET.2003.ETHG028 ; C.ET.2003.ETHG030 ; C.ET.2003.ETHG031 ; C.ET.2003.ETHG033 ; C.ET.2003.ETHG034 ; C.ET.2003.ETHG035 ; C.ET.2003.ETHG036 ; C.ET.2003.ETHG037 ; C.ET.2003.ETHG039 ; C.ET.2003.ETHG046 ; C.ET.2003.ETHG047 ; C.ET.2003.ETHG048 ; C.ET.2003.ETHG050 ; C.ET.2003.ETHG053 ; C.ET.2003.ETHG060 ; C.ET.2003.ETHG066 ; C.ET.2003.ETHG068 ; C.ET.2003.ETHG069 ; C.ET.2003.ETHG071 ; C.ET.2003.ETHG073 ; C.ET.2003.ETHG075 ; C.ET.2003.ETHG077 ; C.ET.2003.ETHG080 ; C.ET.2003.ETHG083 ; C.ET.2003.ETHG086 ; C.ET.2003.ETHG087 ; C.ET.2003.ETHG088 ; C.ET.2003.ETHG089 ; C.ET.2003.ETHG090 ; C.ET.2003.ETHG094 ; C.ET.2003.ETHG097 ; C.ET.2003.ETHG098 ; C.ET.2003.ETHG100 ; C.ET.2003.ETHG108 ; C.ET.2003.ETHG109 ; C.ET.2003.ETHG114 ; C.ET.2003.ETHG116 ; C.ET.2003.ETHG117 ; C.ET.2003.ETHG118 ; C.ET.2003.ETHG123 ; C.ET.2003.ETHG124 ; C.ET.2003.ETHG126 ; C.ET.2003.ETHG129 ; C.ET.2003.ETHG135 ; C.ET.2003.ETHG139 ; C.ET.2003.ETHG140 ; C.ET.2003.ETHG153 ; C.ET.2003.ETHG155 ; C.ET.2003.ETHG159 ; C.ET.2003.ETHG166 ; C.ET.2003.ETHG177 ; C.ET.2003.ETHG180 ; C.ET.2003.ETHG182 ; C.ET.2003.ETHG187 ; C.ET.2003.ETHG201 ; C.ET.2003.ETHG204 ; C.ET.2003.ETHG205 ; C.ET.2003.ETHG208 ; C.ET.2003.ETHG209 ; C.ET.2003.ETHG210 ; C.ET.2003.ETHG216 ; C.ET.2003.ETHG218 ; C.ET.2003.ETHG219 ; C.ET.2003.ETHG225 ; C.ET.2003.ETHG228 ; C.ET.2003.ETHG229 ; C.ET.2003.ETHG232 ; C.ET.2003.ETHG236 ; C.ET.2003.ETHG238 ; C.ET.2003.ETHG239 ; C.ET.2003.ETHG240 ; C.ET.2003.ETHG241 ; C.ET.2003.ETHG242 ; C.ET.2003.ETHG248 ; C.ET.2003.ETHG250 ; C.ET.2003.ETHG251 ; C.ET.2003.ETHG252 ; C.ET.2003.ETHG258 ; C.ET.2003.ETHG260 ; C.GB.2003.118248803220030707 ; C.GB.2003.118248805620030930 ; C.IN.1993.93IN101 ; C.IN.1993.93IN904 ; C.IN.1993.93IN905 ; C.IN.1993.93IN9999 ; C.IN.1994.94IN11246 ; C.IN.1994.94IN206354 ; C.IN.1994.94IN476 ; C.IN.1995.95IN21068 ; C.IN.1998.98IN012 ; C.IN.1998.98IN022 ; C.IN.1999.0185432 ; C.IN.1999.01IN56510 ; C.IN.1999.124662 ; C.IN.1999.160552 ; C.IN.1999.162752 ; C.IN.1999.168452 ; C.IN.1999.169362 ; C.IN.1999.169982 ; C.IN.1999.257102 ; C.IN.1999.257112 ; C.IN.1999.259252 ; C.IN.2000.008362 ; C.IN.2000.RF212392 ; C.IN.2002.YRG36 ; C.IN.2002.YRG46 ; C.IN.2002.YRG49 ; C.IN.2002.YRG50 ; C.IN.2002.YRG6 ; C.IN.2003.D24 ; C.IN.2004.NPT100 ; C.IN.2004.NPT101PL ; C.IN.2004.NPT102 ; C.IN.2004.NPT103PL ; C.IN.2004.NPT104PL ; C.IN.2004.NPT105 ; C.IN.2004.NPT106 ; C.IN.2004.NPT107PL ; C.IN.2004.NPT109PL ; C.IN.2004.NPT11 ; C.IN.2004.NPT110PL ; C.IN.2004.NPT111PL ; C.IN.2004.NPT112PL ; C.IN.2005.G47AFMC27 ; C.IN.2005.G48AFMC34 ; C.IN.2005.G4AFMC2 ; C.IN.2005.G52AFMC28 ; C.IN.2005.G57AFMC29 ; C.IN.2005.G5AFMC3 ; C.IN.2005.G62AFMC35 ; C.IN.2005.G64AFMC36 ; C.IN.2006.CD06406 ; C.IN.2006.CD06411 ; C.IN.2006.CD06415 ; C.IN.2006.CD06419 ; C.IN.2006.CD06420 ; C.IN.2006.CD06444 ; C.IN.2006.CD06468 ; C.IN.2006.CD06469 ; C.IN.2006.CD06481 ; C.IN.2007.cd07005 ; C.IN.2007.VL07332 ; C.IN.2007.VL07337 ; C.IN.2007.VL07347 ; C.IN.2007.VL07355 ; C.IN.2007.VL07363 ; C.IN.2007.VL07365 ; C.IN.2007.VL07366 ; C.IN.2007.VL07368 ; C.IN.2007.VL07381 ; C.IN.2008.HM6 ; C.IN.2008.VL08010 ; C.IN.2008.VL08071 ; C.IN.2008.VL08104 ; C.IN.2008.VL08168 ; C.KE.1991.KNH1268 ; C.KE.1998.98KE384 ; C.KE.2000.KER2010 ; C.TZ.1997.97TZ04 ; C.TZ.1997.97TZ05 ; C.TZ.1998.98TZ013 ; C.TZ.1998.98TZ017 ; C.TZ.2001.A125 ; C.TZ.2001.A207 ; C.TZ.2001.A246 ; C.TZ.2001.A260 ; C.TZ.2001.A301 ; C.TZ.2001.A388 ; C.TZ.2001.A93 ; C.TZ.2001.BD1610 ; C.TZ.2001.BD2211 ; C.TZ.2001.BD398 ; C.TZ.2001.BD911 ; C.TZ.2002.CO178 ; C.TZ.2002.CO3056 ; C.TZ.2002.CO328 ; C.TZ.2002.CO3305 ; C.TZ.2002.CO6130 ; C.TZ.2002.CO6721 ; C.TZ.2005.TBK006 ; C.TZ.2005.TBK009 ; C.TZ.2005.TBK019 ; C.TZ.2005.TBK036 ; C.TZ.2005.TBK046 ; C.TZ.2005.TBK049 ; C.TZ.2005.TBK052 ; C.TZ.2005.TBK055 ; C.TZ.2005.TBK063 ; C.TZ.2005.TBK070 ; C.TZ.2005.TBK071 ; C.TZ.2005.TBK073 ; C.TZ.2005.TBK075 ; C.TZ.2005.TBK076 ; C.TZ.2005.TBK077 ; C.TZ.2005.TBK086 ; C.TZ.2005.TBK094 ; C.TZ.2005.TBK096 ; C.TZ.2005.TMS009 ; C.TZ.2005.TMS012 ; C.TZ.2005.TMS020 ; C.TZ.2005.TMS028 ; C.TZ.2005.TMS029 ; C.TZ.2005.TMS031 ; C.TZ.2005.TMS101 ; C.TZ.2005.TMS102 ; C.TZ.2005.TMS114 ; C.TZ.2005.TMS118 ; C.TZ.2005.TMS125 ; C.TZ.2005.TMS128 ; C.TZ.2005.TMS130 ; C.TZ.2005.TMS133 ; C.TZ.2005.TMS202 ; C.TZ.2005.TMS205 ; C.TZ.2005.TMS208 ; C.TZ.2005.TMS211 ; C.TZ.2005.TMS214 ; C.TZ.2005.TMS216 ; C.TZ.2005.TMS223 ; C.TZ.2005.TMS316 ; C.TZ.2005.TMS321 ; C.TZ.2005.TMS324 ; C.TZ.2005.TMS326 ; C.TZ.2005.TMS348 ; C.UG.121108V ; C.UG.1990.UG268 ; C.UG.1997.513 ; C.UG.1997.541 ; C.UG.1997.613 ; C.UG.1997.681 ; C.UG.1997.73 ; C.UG.1997.823 ; C.UG.1997.840 ; C.UG.2002.TC023004 ; C.UG.2006.IAEAM020 ; C.ZA.1997.97ZA003 ; C.ZA.1997.97ZA009 ; C.ZA.1997.97ZA012 ; C.ZA.1998.98TV002 ; C.ZA.1998.98ZA445 ; C.ZA.1998.98ZA502 ; C.ZA.1998.98ZA528 ; C.ZA.1998.CTSC2 ; C.ZA.1998.DU151p2 ; C.ZA.1998.TV001 ; C.ZA.1998.TV012 ; C.ZA.1999.99ZACM9 ; C.ZA.1999.99ZATM10 ; C.ZA.1999.DU179 ; C.ZA.1999.DU422 ; C.ZA.1999.ZASW7 ; C.ZA.2000.00ZADR5 ; C.ZA.2000.00ZADR6 ; C.ZA.2000.1119MB ; C.ZA.2000.1134MB ; C.ZA.2000.1157M3M ; C.ZA.2000.1162MB ; C.ZA.2000.1165MB ; C.ZA.2000.1168MB ; C.ZA.2000.1170MB ; C.ZA.2000.1171MB ; C.ZA.2000.1176MB ; C.ZA.2000.1178MB ; C.ZA.2000.1180 ; C.ZA.2000.1192M3M ; C.ZA.2000.1195MB ; C.ZA.2000.1197MB ; C.ZA.2000.1199 ; C.ZA.2000.1200 ; C.ZA.2000.1210MB ; C.ZA.2000.1214MB ; C.ZA.2000.1217MB ; C.ZA.2000.1225MB ; C.ZA.2000.1228MB ; C.ZA.2000.CZA1069MB ; C.ZA.2000.CZA1184MB ; C.ZA.2000.CZA1189MB ; C.ZA.2000.CZAJ112MA ; C.ZA.2000.J38MA ; C.ZA.2001.01ZATM45 ; C.ZA.2001.2006 ; C.ZA.2001.2110 ; C.ZA.2001.2134MB ; C.ZA.2001.2344 ; C.ZA.2001.2592 ; C.ZA.2001.J54Ma ; C.ZA.2001.ZA001P01 ; C.ZA.2001.ZA002P01 ; C.ZA.2001.ZA003P01 ; C.ZA.2001.ZA004P01 ; C.ZA.2001.ZA005P01 ; C.ZA.2001.ZA006P01 ; C.ZA.2001.ZA007P01 ; C.ZA.2001.ZA008P01 ; C.ZA.2001.ZA009P01 ; C.ZA.2001.ZA010P01 ; C.ZA.2001.ZA011P01 ; C.ZA.2001.ZA012P01 ; C.ZA.2001.ZA013P01 ; C.ZA.2001.ZA014P01 ; C.ZA.2001.ZA015P01 ; C.ZA.2001.ZA016P01 ; C.ZA.2001.ZA017P01 ; C.ZA.2001.ZA018P01 ; C.ZA.2001.ZA019P01 ; C.ZA.2001.ZA020P01 ; C.ZA.2001.ZA021P01 ; C.ZA.2001.ZA022P01 ; C.ZA.2001.ZA023P01 ; C.ZA.2001.ZA024P01 ; C.ZA.2001.ZA025P01 ; C.ZA.2001.ZA026P01 ; C.ZA.2001.ZA027P01 ; C.ZA.2001.ZA028P01 ; C.ZA.2001.ZA029P01 ; C.ZA.2001.ZA030P01 ; C.ZA.2001.ZA031P01 ; C.ZA.2001.ZA032P01 ; C.ZA.2001.ZA033P01 ; C.ZA.2001.ZA034P01 ; C.ZA.2001.ZA035P01 ; C.ZA.2001.ZA036P01 ; C.ZA.2001.ZA037P01 ; C.ZA.2001.ZA038P01 ; C.ZA.2001.ZA039P01 ; C.ZA.2001.ZA041P01 ; C.ZA.2001.ZA042P01 ; C.ZA.2001.ZA043P01 ; C.ZA.2001.ZA044P01 ; C.ZA.2001.ZA045P01 ; C.ZA.2001.ZA046P01 ; C.ZA.2001.ZA047p01 ; C.ZA.2001.ZA048p01 ; C.ZA.2001.ZA049p01 ; C.ZA.2001.ZA050p01 ; C.ZA.2001.ZA051p01 ; C.ZA.2001.ZA052p01 ; C.ZA.2001.ZA053p01 ; C.ZA.2001.ZA054p01 ; C.ZA.2001.ZA055p01 ; C.ZA.2001.ZA057p01 ; C.ZA.2001.ZA058p01 ; C.ZA.2001.ZA059p01 ; C.ZA.2001.ZA060p01 ; C.ZA.2001.ZA061p01 ; C.ZA.2001.ZA062p01 ; C.ZA.2001.ZA063P01 ; C.ZA.2001.ZA064P01 ; C.ZA.2001.ZA065P01 ; C.ZA.2001.ZA066P01 ; C.ZA.2001.ZA068p01 ; C.ZA.2001.ZA069p01 ; C.ZA.2001.ZA071P02 ; C.ZA.2001.ZA073p01 ; C.ZA.2001.ZA074p01 ; C.ZA.2001.ZA075p01 ; C.ZA.2001.ZA077P02 ; C.ZA.2001.ZA078P02 ; C.ZA.2001.ZA080P01 ; C.ZA.2001.ZA40p ; C.ZA.2002.02ZADR10 ; C.ZA.2002.02ZADR11 ; C.ZA.2002.02ZADR13 ; C.ZA.2002.02ZADR14 ; C.ZA.2002.02ZADR15 ; C.ZA.2002.02ZADR16 ; C.ZA.2002.02ZADR18 ; C.ZA.2002.02ZADR19 ; C.ZA.2002.02ZADR20 ; C.ZA.2002.02ZADR23 ; C.ZA.2002.02ZADR27 ; C.ZA.2002.02ZADR28 ; C.ZA.2002.02ZADR29 ; C.ZA.2002.02ZADR30 ; C.ZA.2002.02ZADR31 ; C.ZA.2002.02ZADR32 ; C.ZA.2002.02ZADR33 ; C.ZA.2002.02ZADR34 ; C.ZA.2002.02ZADR35 ; C.ZA.2002.02ZADR36 ; C.ZA.2002.02ZADR37 ; C.ZA.2002.02ZADR39 ; C.ZA.2002.02ZADR41 ; C.ZA.2002.02ZADR42 ; C.ZA.2002.02ZADR43 ; C.ZA.2002.02ZADR44 ; C.ZA.2002.02ZADR46 ; C.ZA.2002.02ZADR47 ; C.ZA.2002.02ZADR49 ; C.ZA.2002.02ZADR50 ; C.ZA.2002.02ZADR51 ; C.ZA.2002.02ZADR52 ; C.ZA.2002.02ZADR53 ; C.ZA.2002.02ZADR54 ; C.ZA.2002.02ZADR55 ; C.ZA.2002.02ZADR56 ; C.ZA.2002.02ZADR58 ; C.ZA.2002.02ZADR60 ; C.ZA.2002.02ZADR61 ; C.ZA.2002.02ZADR64 ; C.ZA.2002.02ZADR66 ; C.ZA.2002.02ZADR67 ; C.ZA.2002.02ZADR68 ; C.ZA.2002.02ZADR69 ; C.ZA.2002.02ZADR70 ; C.ZA.2002.02ZADR71 ; C.ZA.2002.02ZAPS001MB1 ; C.ZA.2002.02ZAPS005MB1 ; C.ZA.2002.02ZAPS006MB1 ; C.ZA.2002.02ZAPS008MB1 ; C.ZA.2002.02ZAPS013MB1 ; C.ZA.2002.02ZAPS014MB1 ; C.ZA.2002.02ZAPS015MB1 ; C.ZA.2002.1043C ; C.ZA.2002.1043M ; C.ZA.2002.1156 ; C.ZA.2002.1440 ; C.ZA.2002.1446 ; C.ZA.2002.1448 ; C.ZA.2002.1450 ; C.ZA.2002.1484B ; C.ZA.2002.1514B ; C.ZA.2002.1613 ; C.ZA.2002.1614 ; C.ZA.2002.1666 ; C.ZA.2002.1668 ; C.ZA.2002.1676 ; C.ZA.2002.1677 ; C.ZA.2002.1680 ; C.ZA.2002.1697 ; C.ZA.2002.1742 ; C.ZA.2002.1743 ; C.ZA.2002.1744 ; C.ZA.2002.1745 ; C.ZA.2002.1748 ; C.ZA.2002.1749 ; C.ZA.2002.1752 ; C.ZA.2002.1753 ; C.ZA.2002.1754 ; C.ZA.2002.1755 ; C.ZA.2002.1756 ; C.ZA.2002.1757 ; C.ZA.2002.1758 ; C.ZA.2002.1759 ; C.ZA.2002.1760 ; C.ZA.2002.1761 ; C.ZA.2002.1762 ; C.ZA.2002.1763 ; C.ZA.2002.1764 ; C.ZA.2002.1767 ; C.ZA.2002.1768 ; C.ZA.2002.1770 ; C.ZA.2002.1771 ; C.ZA.2002.1772 ; C.ZA.2002.1773 ; C.ZA.2002.1774 ; C.ZA.2002.1775 ; C.ZA.2002.1777 ; C.ZA.2002.1778 ; C.ZA.2002.1779 ; C.ZA.2002.1780 ; C.ZA.2002.1781 ; C.ZA.2002.1782 ; C.ZA.2002.1783 ; C.ZA.2002.1785 ; C.ZA.2002.1786 ; C.ZA.2002.1787 ; C.ZA.2002.1790 ; C.ZA.2002.1792 ; C.ZA.2002.1795 ; C.ZA.2002.1796 ; C.ZA.2002.1799 ; C.ZA.2002.1800 ; C.ZA.2002.1802 ; C.ZA.2002.1803 ; C.ZA.2002.1804 ; C.ZA.2002.1805 ; C.ZA.2002.1806 ; C.ZA.2002.1807 ; C.ZA.2002.1809 ; C.ZA.2002.1813 ; C.ZA.2002.1814 ; C.ZA.2002.1815 ; C.ZA.2002.1816 ; C.ZA.2002.1818 ; C.ZA.2002.1820 ; C.ZA.2002.1821 ; C.ZA.2002.1823 ; C.ZA.2002.1824 ; C.ZA.2002.1831 ; C.ZA.2002.1832 ; C.ZA.2002.PS003 ; C.ZA.2002.PS007 ; C.ZA.2002.PS012 ; C.ZA.2002.ZA079P02 ; C.ZA.2003.03ZADR100 ; C.ZA.2003.03ZADR102 ; C.ZA.2003.03ZADR74 ; C.ZA.2003.03ZADR76 ; C.ZA.2003.03ZADR79 ; C.ZA.2003.03ZADR80 ; C.ZA.2003.03ZADR81 ; C.ZA.2003.03ZADR82 ; C.ZA.2003.03ZADR84 ; C.ZA.2003.03ZADR88 ; C.ZA.2003.03ZADR92 ; C.ZA.2003.03ZADR93 ; C.ZA.2003.03ZADR94 ; C.ZA.2003.03ZADR95 ; C.ZA.2003.03ZADR98 ; C.ZA.2003.03ZAPS017MB1 ; C.ZA.2003.03ZAPS020MB1 ; C.ZA.2003.03ZAPS021MB1 ; C.ZA.2003.03ZAPS023MB1 ; C.ZA.2003.03ZAPS024MB1 ; C.ZA.2003.03ZAPS025MB1 ; C.ZA.2003.03ZAPS026MB1 ; C.ZA.2003.03ZAPS027MB1 ; C.ZA.2003.03ZAPS030MB1 ; C.ZA.2003.03ZAPS032MB1 ; C.ZA.2003.03ZAPS034MB1 ; C.ZA.2003.03ZAPS042MB1 ; C.ZA.2003.03ZAPS043MB1 ; C.ZA.2003.03ZAPS044MB1 ; C.ZA.2003.03ZAPS046MB1 ; C.ZA.2003.03ZAPS048MB1 ; C.ZA.2003.03ZAPS049MB1 ; C.ZA.2003.03ZAPS050MB1 ; C.ZA.2003.03ZAPS051MB1 ; C.ZA.2003.03ZAPS052MB1 ; C.ZA.2003.03ZAPS054MB2 ; C.ZA.2003.03ZAPS055MB1 ; C.ZA.2003.03ZAPS056MB1 ; C.ZA.2003.03ZAPS057MB2 ; C.ZA.2003.03ZAPS059MB2 ; C.ZA.2003.03ZAPS063MB1 ; C.ZA.2003.03ZAPS066MB2 ; C.ZA.2003.03ZAPS067MB2 ; C.ZA.2003.03ZAPS071MB1 ; C.ZA.2003.03ZAPS073MB1 ; C.ZA.2003.03ZAPS074MB2 ; C.ZA.2003.03ZAPS077B1 ; C.ZA.2003.03ZAPS079B1 ; C.ZA.2003.03ZAPS081MB1 ; C.ZA.2003.03ZAPS083MB1 ; C.ZA.2003.03ZAPS086MB1 ; C.ZA.2003.03ZAPS088MB1 ; C.ZA.2003.03ZAPS089MB1 ; C.ZA.2003.03ZAPS091MB1 ; C.ZA.2003.03ZAPS094MB1 ; C.ZA.2003.03ZAPS095MB1 ; C.ZA.2003.03ZAPS097MB1 ; C.ZA.2003.03ZAPS099MB1 ; C.ZA.2003.03ZAPS103MB2 ; C.ZA.2003.03ZAPS104MB1 ; C.ZA.2003.03ZAPS105MB2 ; C.ZA.2003.03ZAPS108MB1 ; C.ZA.2003.03ZAPS112MB2 ; C.ZA.2003.03ZAPS113MB2 ; C.ZA.2003.03ZAPS116MB1 ; C.ZA.2003.03ZAPS118MB1 ; C.ZA.2003.03ZAPS11MB2 ; C.ZA.2003.03ZAPS122MB1 ; C.ZA.2003.03ZAPS123MB1 ; C.ZA.2003.03ZAPS124MB1 ; C.ZA.2003.03ZAPS125MB1 ; C.ZA.2003.03ZAPS126MB1 ; C.ZA.2003.03ZAPS128MB1 ; C.ZA.2003.03ZAPS130MB1 ; C.ZA.2003.03ZAPS131MB1 ; C.ZA.2003.03ZAPS133MB1 ; C.ZA.2003.03ZAPS136MB1 ; C.ZA.2003.03ZAPS140MB1 ; C.ZA.2003.03ZAPS143MB1 ; C.ZA.2003.03ZAPS151MB1 ; C.ZA.2003.03ZAPS152MB1 ; C.ZA.2003.03ZAPS155MB1 ; C.ZA.2003.03ZASK005B2 ; C.ZA.2003.03ZASK006B2 ; C.ZA.2003.03ZASK010B2 ; C.ZA.2003.03ZASK011B2 ; C.ZA.2003.03ZASK013B2 ; C.ZA.2003.03ZASK019B2 ; C.ZA.2003.03ZASK020B2 ; C.ZA.2003.03ZASK026B2 ; C.ZA.2003.03ZASK034B1 ; C.ZA.2003.03ZASK036B1 ; C.ZA.2003.03ZASK039B2 ; C.ZA.2003.03ZASK058B2 ; C.ZA.2003.03ZASK061B1 ; C.ZA.2003.03ZASK062B1 ; C.ZA.2003.03ZASK066B1 ; C.ZA.2003.03ZASK067B1 ; C.ZA.2003.03ZASK072B1 ; C.ZA.2003.03ZASK073B1 ; C.ZA.2003.03ZASK076B1 ; C.ZA.2003.03ZASK078B1 ; C.ZA.2003.03ZASK084B1 ; C.ZA.2003.03ZASK092B1 ; C.ZA.2003.03ZASK094B1 ; C.ZA.2003.03ZASK097B1 ; C.ZA.2003.03ZASK098B1 ; C.ZA.2003.03ZASK103B1 ; C.ZA.2003.03ZASK104B1 ; C.ZA.2003.03ZASK107B1 ; C.ZA.2003.03ZASK110B1 ; C.ZA.2003.03ZASK111B1 ; C.ZA.2003.03ZASK113B1 ; C.ZA.2003.03ZASK117B1 ; C.ZA.2003.03ZASK118B1 ; C.ZA.2003.03ZASK120B1 ; C.ZA.2003.03ZASK211B1 ; C.ZA.2003.03ZASK212B1 ; C.ZA.2003.03ZASK213B1 ; C.ZA.2003.03ZASK215M6W ; C.ZA.2003.03ZASK223B1 ; C.ZA.2003.03ZASK224MB1 ; C.ZA.2003.03ZASK226B1 ; C.ZA.2003.03ZASK232B1 ; C.ZA.2003.03ZASK233B1 ; C.ZA.2003.04ZAPS016MB1 ; C.ZA.2003.04ZAPS194MB1 ; C.ZA.2003.04ZASK082B1 ; C.ZA.2003.PS029 ; C.ZA.2003.PS037 ; C.ZA.2003.PS045 ; C.ZA.2003.PS047 ; C.ZA.2003.PS058 ; C.ZA.2003.PS060 ; C.ZA.2003.PS087 ; C.ZA.2003.PS093 ; C.ZA.2003.PS096 ; C.ZA.2003.PS098 ; C.ZA.2003.PS101 ; C.ZA.2003.PS106 ; C.ZA.2003.PS109 ; C.ZA.2003.PS111 ; C.ZA.2003.PS117 ; C.ZA.2003.PS119 ; C.ZA.2003.PS120 ; C.ZA.2003.PS141 ; C.ZA.2003.PS145 ; C.ZA.2003.PS147 ; C.ZA.2003.PS150 ; C.ZA.2003.PS156 ; C.ZA.2003.SK023B2 ; C.ZA.2003.SK029B2 ; C.ZA.2003.SK033B2 ; C.ZA.2003.SK040B1 ; C.ZA.2003.SK041B1 ; C.ZA.2003.SK043B1 ; C.ZA.2003.SK065B1 ; C.ZA.2003.SK112B1 ; C.ZA.2003.SK116B1 ; C.ZA.2004.04ZAPS157MB1 ; C.ZA.2004.04ZAPS160B1 ; C.ZA.2004.04ZAPS161B1 ; C.ZA.2004.04ZAPS165MB1 ; C.ZA.2004.04ZAPS168B1 ; C.ZA.2004.04ZAPS169MB1 ; C.ZA.2004.04ZAPS172MB1 ; C.ZA.2004.04ZAPS177MB1 ; C.ZA.2004.04ZAPS188B1 ; C.ZA.2004.04ZAPS189B1 ; C.ZA.2004.04ZAPS190B1 ; C.ZA.2004.04ZAPS193B1 ; C.ZA.2004.04ZAPS195B1 ; C.ZA.2004.04ZAPS197MB1 ; C.ZA.2004.04ZAPS198MB1 ; C.ZA.2004.04ZAPS199B1 ; C.ZA.2004.04ZAPS201B1 ; C.ZA.2004.04ZAPS202B1 ; C.ZA.2004.04ZAPS205B1 ; C.ZA.2004.04ZAPS206B1 ; C.ZA.2004.04ZAPS214B1 ; C.ZA.2004.04ZAPS216B1 ; C.ZA.2004.04ZAPS217B1 ; C.ZA.2004.04ZASK031B2 ; C.ZA.2004.04ZASK083B2 ; C.ZA.2004.04ZASK127B1 ; C.ZA.2004.04ZASK128B1 ; C.ZA.2004.04ZASK131B1 ; C.ZA.2004.04ZASK132B1 ; C.ZA.2004.04ZASK135B1 ; C.ZA.2004.04ZASK136B1 ; C.ZA.2004.04ZASK139B1 ; C.ZA.2004.04ZASK142B1 ; C.ZA.2004.04ZASK145B1 ; C.ZA.2004.04ZASK147B1 ; C.ZA.2004.04ZASK148B1 ; C.ZA.2004.04ZASK150B1 ; C.ZA.2004.04ZASK151B1 ; C.ZA.2004.04ZASK154B1 ; C.ZA.2004.04ZASK155B1 ; C.ZA.2004.04ZASK156B1 ; C.ZA.2004.04ZASK159B1 ; C.ZA.2004.04ZASK160B1 ; C.ZA.2004.04ZASK161B1 ; C.ZA.2004.04ZASK163B1 ; C.ZA.2004.04ZASK164B1 ; C.ZA.2004.04ZASK165B1 ; C.ZA.2004.04ZASK167B1 ; C.ZA.2004.04ZASK168B1 ; C.ZA.2004.04ZASK169B1 ; C.ZA.2004.04ZASK170B1 ; C.ZA.2004.04ZASK171B1 ; C.ZA.2004.04ZASK173B1 ; C.ZA.2004.04ZASK174B1 ; C.ZA.2004.04ZASK175B1 ; C.ZA.2004.04ZASK176B1 ; C.ZA.2004.04ZASK178B1 ; C.ZA.2004.04ZASK180B1 ; C.ZA.2004.04ZASK181B1 ; C.ZA.2004.04ZASK182B1 ; C.ZA.2004.04ZASK183B1 ; C.ZA.2004.04ZASK184B1 ; C.ZA.2004.04ZASK185B1 ; C.ZA.2004.04ZASK190B1 ; C.ZA.2004.04ZASK191B1 ; C.ZA.2004.04ZASK192B1 ; C.ZA.2004.04ZASK196B1 ; C.ZA.2004.04ZASK200B1 ; C.ZA.2004.04ZASK202B1 ; C.ZA.2004.04ZASK204B1 ; C.ZA.2004.04ZASK206B1 ; C.ZA.2004.04ZASK208B1 ; C.ZA.2004.04ZASK217B1 ; C.ZA.2004.04ZASK234B1 ; C.ZA.2004.PS159 ; C.ZA.2004.PS171 ; C.ZA.2004.PS174 ; C.ZA.2004.PS175 ; C.ZA.2004.PS178 ; C.ZA.2004.PS182 ; C.ZA.2004.PS186 ; C.ZA.2004.PS187 ; C.ZA.2004.PS191 ; C.ZA.2004.PS192 ; C.ZA.2004.PS203 ; C.ZA.2004.PS204 ; C.ZA.2004.PS208 ; C.ZA.2004.PS209 ; C.ZA.2004.PS210 ; C.ZA.2004.PS212 ; C.ZA.2004.SK133B1 ; C.ZA.2004.SK134B1 ; C.ZA.2004.SK140B1 ; C.ZA.2004.SK143B1 ; C.ZA.2004.SK144B1 ; C.ZA.2004.SK164B1 ; C.ZA.2005.05ZAPSK240B1 ; C.ZA.2005.05ZASK243B1 ; C.ZA.2005.05ZASK244B1 ; C.ZA.2005.05ZASK245B1 ; C.ZA.2005.05ZASK246B1 ; C.ZA.2005.05ZASK247B1 ; C.ZA.2005.41R ; C.ZA.2006.06ZACHB115B ; C.ZA.2006.06ZACHB115M ; C.ZA.2006.06ZACHB142B ; C.ZA.2006.06ZACHB142M ; C.ZA.2006.06ZACHB147B ; C.ZA.2006.06ZACHB147M ; C.ZA.2006.06ZACHB188B ; C.ZA.2006.06ZACHB188M ; C.ZA.2006.06ZACHB200B ; C.ZA.2006.06ZACHB200M ; C.ZA.2006.06ZACHB226B ; C.ZA.2006.06ZACHB226M ; C.ZA.2006.06ZACHB231B ; C.ZA.2006.06ZACHB231M ; C.ZA.2006.06ZACHB253B ; C.ZA.2006.06ZACHB253M ; C.ZA.2006.06ZACHB265B ; C.ZA.2006.06ZACHB265M ; C.ZA.2006.06ZACHB309B ; C.ZA.2006.06ZACHB309M ; C.ZA.2006.06ZACHB374B ; C.ZA.2006.06ZACHB374M ; C.ZA.2006.06ZACHB398B ; C.ZA.2006.06ZACHB398M ; C.ZA.2006.06ZACHB4B ; C.ZA.2006.06ZACHB4M ; C.ZA.2006.06ZACHB5B ; C.ZA.2006.06ZACHB5M ; C.ZA.2006.06ZACHB64B ; C.ZA.2006.06ZACHB64M ; C.ZA.2006.06ZACHB83B ; C.ZA.2006.06ZACHB83M ; C.ZA.2006.06ZAKEH109B ; C.ZA.2006.06ZAKEH109M ; C.ZA.2006.06ZAKEH142B ; C.ZA.2006.06ZAKEH142M ; C.ZA.2006.06ZAKEH158B ; C.ZA.2006.06ZAKEH158M ; C.ZA.2006.06ZAKEH15B ; C.ZA.2006.06ZAKEH15M ; C.ZA.2006.06ZAKEH161B ; C.ZA.2006.06ZAKEH161M ; C.ZA.2006.06ZAKEH165B ; C.ZA.2006.06ZAKEH165M ; C.ZA.2006.06ZAKEH169B ; C.ZA.2006.06ZAKEH169M ; C.ZA.2006.06ZAKEH181B ; C.ZA.2006.06ZAKEH181M ; C.ZA.2006.06ZAKEH205B ; C.ZA.2006.06ZAKEH205M ; C.ZA.2006.06ZAKEH210B ; C.ZA.2006.06ZAKEH210M ; C.ZA.2006.06ZAKEH2B ; C.ZA.2006.06ZAKEH2M ; C.ZA.2006.06ZAKEH32B ; C.ZA.2006.06ZAKEH32M ; C.ZA.2006.06ZAKEH44B ; C.ZA.2006.06ZAKEH44M ; C.ZA.2006.06ZAKEH56B ; C.ZA.2006.06ZAKEH56M ; C.ZA.2006.06ZAKEH62B ; C.ZA.2006.06ZAKEH62M ; C.ZA.2006.06ZAKEH79B ; C.ZA.2006.06ZAKEH79M ; C.ZA.2006.102R ; C.ZA.2006.112R ; C.ZA.2006.11R ; C.ZA.2006.121R ; C.ZA.2006.122R ; C.ZA.2006.12R ; C.ZA.2006.131R ; C.ZA.2006.132R ; C.ZA.2006.142R ; C.ZA.2006.161R ; C.ZA.2006.171R ; C.ZA.2006.172R ; C.ZA.2006.173R ; C.ZA.2006.191R ; C.ZA.2006.201R ; C.ZA.2006.211R ; C.ZA.2006.212R ; C.ZA.2006.21R ; C.ZA.2006.222R ; C.ZA.2006.223R ; C.ZA.2006.231R ; C.ZA.2006.232R ; C.ZA.2006.252R ; C.ZA.2006.271R ; C.ZA.2006.281R ; C.ZA.2006.31R ; C.ZA.2006.42R ; C.ZA.2006.61R ; C.ZA.2006.62R ; C.ZA.2006.71R ; C.ZA.2006.82R ; C.ZA.2006.83R ; C.ZA.2006.91R ; C.ZA.2007.103R ; C.ZA.2007.104R ; C.ZA.2007.113R ; C.ZA.2007.114R ; C.ZA.2007.123R ; C.ZA.2007.124R ; C.ZA.2007.133R ; C.ZA.2007.13R ; C.ZA.2007.143R ; C.ZA.2007.152R ; C.ZA.2007.153R ; C.ZA.2007.162R ; C.ZA.2007.163R ; C.ZA.2007.182R ; C.ZA.2007.183R ; C.ZA.2007.192R ; C.ZA.2007.193R ; C.ZA.2007.202R ; C.ZA.2007.203R ; C.ZA.2007.213R ; C.ZA.2007.224R ; C.ZA.2007.22R ; C.ZA.2007.233R ; C.ZA.2007.234R ; C.ZA.2007.23R ; C.ZA.2007.242R ; C.ZA.2007.243R ; C.ZA.2007.253R ; C.ZA.2007.272R ; C.ZA.2007.273R ; C.ZA.2007.282R ; C.ZA.2007.283R ; C.ZA.2007.32R ; C.ZA.2007.44R ; C.ZA.2007.45R ; C.ZA.2007.63R ; C.ZA.2007.72R ; C.ZA.2007.73R ; C.ZA.2007.84R ; C.ZA.2007.93R ; C.ZA.2007.94R ; C.ZA.99ZALT21 ; C.ZA.99ZALT39 ; C.ZA.99ZALT4 ; C.ZA.99ZALT42 ; C.ZA.99ZALT45 ; C.ZA.99ZALT46 ; C.ZA.99ZALT5 ;

**UK HIV Drug Resistance Database sequence IDs**

nUK100092 ; nUK100213 ; nUK100277 ; nUK100515 ; nUK100869 ; nUK101149 ; nUK101867 ; nUK102474 ; nUK104190 ; nUK104218 ; nUK104593 ; nUK105313 ; nUK105793 ; nUK105898 ; nUK106190 ; nUK106197 ; nUK106433 ; nUK106984 ; nUK107220 ; nUK107588 ; nUK107637 ; nUK108186 ; nUK108397 ; nUK108944 ; nUK109123 ; nUK109846 ; nUK110239 ; nUK110878 ; nUK111188 ; nUK112206 ; nUK112427 ; nUK113432 ; nUK113971 ; nUK114174 ; nUK114380 ; nUK114742 ; nUK114778 ; nUK114959 ; nUK115022 ; nUK115545 ; nUK115841 ; nUK115896 ; nUK115982 ; nUK116233 ; nUK116320 ; nUK117012 ; nUK117307 ; nUK117418 ; nUK117562 ; nUK118024 ; nUK118403 ; nUK118541 ; nUK118730 ; nUK118741 ; nUK119132 ; nUK119178 ; nUK119374 ; nUK120024 ; nUK120396 ; nUK120966 ; nUK121133 ; nUK122327 ; nUK123028 ; nUK79480 ; nUK79646 ; nUK79726 ; nUK79903 ; nUK80095 ; nUK80167 ; nUK80317 ; nUK80422 ; nUK80502 ; nUK80704 ; nUK80900 ; nUK80914 ; nUK81169 ; nUK81380 ; nUK81470 ; nUK81881 ; nUK82118 ; nUK82315 ; nUK82346 ; nUK82644 ; nUK83188 ; nUK83526 ; nUK83698 ; nUK84291 ; nUK84378 ; nUK84582 ; nUK84720 ; nUK84835 ; nUK86200 ; nUK86808 ; nUK86861 ; nUK86969 ; nUK87145 ; nUK87175 ; nUK87562 ; nUK87592 ; nUK87821 ; nUK87894 ; nUK87950 ; nUK88244 ; nUK88338 ; nUK88342 ; nUK88368 ; nUK88370 ; nUK88640 ; nUK89036 ; nUK89985 ; nUK90539 ; nUK90567 ; nUK90617 ; nUK90963 ; nUK91117 ; nUK91156 ; nUK91705 ; nUK91975 ; nUK92256 ; nUK92377 ; nUK92546 ; nUK92669 ; nUK93569 ; nUK93814 ; nUK94580 ; nUK94623 ; nUK95146 ; nUK95212 ; nUK96261 ; nUK97117 ; nUK97406 ; nUK97772 ; nUK98151 ; nUK98259 ; nUK98315 ; nUK98519 ; nUK98795 ; nUK99087
